# Effects of the Reactive Moiety of Phenolipids on Their Antioxidant Efficiency in Model Emulsified Systems

**DOI:** 10.3390/foods10051028

**Published:** 2021-05-10

**Authors:** Marlene Costa, Sonia Losada-Barreiro, Júlia Magalhães, Luís S. Monteiro, Carlos Bravo-Díaz, Fátima Paiva-Martins

**Affiliations:** 1 REQUIMTE/LAQV, Department of Chemistry and Biochemistry, Faculty of Sciences, University of Porto, Campo Alegre 687, 4169-007 Porto, Portugal; marlene.andreia.costa@gmail.com (M.C.); sonia@uvigo.es (S.L.-B.); 2 Department of Physical-Chemistry, Faculty of Chemistry, Universidade de Vigo, 36310 Vigo, Spain; cbravo@uvigo.es; 3 REQUIMTE/LAQV, Department of Chemical Engineering, Faculty of Engineering, University of Porto, 4200-465 Porto, Portugal; jmagalh@fe.up.pt; 4 Chemistry Centre, University of Minho, Gualtar, 4710-057 Braga, Portugal; monteiro@quimica.uminho.pt

**Keywords:** antioxidants, emulsions, caffeic acid, dihydrocaffeic acid, hydroxytyrosol, oxidative stability, interfacial concentration, catechols

## Abstract

Our previous research was focused on the effects of hydrophobicity on the antioxidant (AO) efficiency of series of homologous antioxidants with the same reactive moieties. In this work we evaluate the antioxidant efficiency of hydrophobic phenolipids in 4:6 olive oil-in-water emulsions, with different phenolic moieties (derived from caffeic, 4-hydroxycinnamic, dihydrocaffeic acids, tyrosol and hydroxytyrosol), with alkyl chains of 8 and 16 carbons, and compare the antioxidant efficiency with that of the parent compounds. All catecholic phenolipids, in particular the C8 derivatives, have proven to be better antioxidants for the oxidative protection of emulsions than their parental compounds with octyl dihydrocafffeate being the most efficient (16-fold increase in relation to the control). To understand the importance of some factors on the antioxidant efficiency of compounds in emulsions, Pearson’s correlation analysis was carried out between antioxidant activity and the first anodic potential (*E*_pa_), reducing capacity (FRAP value), DPPH radical scavenging activity (EC_50_) and the concentration of antioxidants in each region of the emulsified system. Results confirm the importance of the effective concentration of AOs in the interfacial region (AO_I_) (ρ = 0.820) and of the *E*_pa_ (ρ = −0.677) in predicting their antioxidant efficiency in olive oil-in-water emulsions.

## 1. Introduction

Oil-in-water (O/W) emulsions are widely used not only in the food industry but also in the cosmetic, pharmaceutical and medical industries to encapsulate, protect and release bioactive lipids and lipophilic molecules [1]. However, the industry faces a serious problem due to the low oxidative stability of emulsified systems, which compromises sensory properties and organoleptic characteristics, consumer safety and economic viability of products. Thus, in order to retard or inhibit the oxidative process, and increase the health aspect and nutritional value of foods, addition of an antioxidant agent in these systems is a very appealing strategy. This is due to their ease of use and economy of the process when compared to other strategies such as the use of a specific package developed for the purpose, reduction of oxygen pressure in the package or the use of very low storage temperatures (below −20 °C). However, lipid oxidation and antioxidant (AO) mechanisms in emulsified systems are complex phenomena. Numerous variables can influence the rate and extent of lipid oxidation and the effectiveness of antioxidants. The ability of antioxidants to inhibit lipid oxidation in emulsions depends on factors such as the antioxidant concentration, reactivity, partitioning between oil, water and interfacial regions, interactions with other emulsion components such as emulsifiers, other AOs and environmental conditions such as pH, ionic strength and temperature [2]. Therefore, with the goal of developing methods to control the negative impact on their oxidative stability, numerous studies have been conducted in order to understand the lipid oxidation and its inhibition process in oil-in-water emulsions [3].

One of the most important parameters that influences lipid oxidation in emulsions is the degree of unsaturation of the fatty acids of the oil phase. Oxidative deterioration of highly unsaturated edible oils such as fish oil, is one of the most important problems in the field of food chemistry, because lipid oxidation products not only cause undesirable flavors but also decrease the nutritional quality and safety of lipid-containing foods. The oxidation rate of polyunsaturated fatty acids (PUFAs) is directly proportional to the number of bis-allylic hydrogens present in their molecules [4]. Therefore, it is generally accepted that fish oils, containing high levels of n-3 PUFAs such as docosahexaenoic acid (DHA, 22:6n-3) and eicosapentaenoic acid (EPA, 20:5n-3), are highly susceptible to autoxidation. Despite its high value in omega-3 fatty acids, fish oil is considered a by-product of fisheries. Every year tons of this oil are not used as a food ingredient because of its very low oxidative stability and high costs in purification in order to be safe for human consumption. Therefore, the need for better antioxidants and the need for understanding how the antioxidant activity of compounds is affected by several factors in food systems, has been mainly pulled by the need of stabilizing highly oxidative unstable oils such as fish oil. 

As fish oil oxidative stability is low, it is often difficult to assess which factors influence the antioxidant activity of AOs in emulsified systems containing this type of oil. Therefore, in the present study, we use a more stable oil, stripped olive oil, in the evaluation of the antioxidant efficiency of several different structured phenolipids and their parental compounds in O/W emulsions in order to be able to clearly assess the differences in their antioxidant efficiency. Moreover, we aim to correlate these differences in the antioxidant capacity with some of AOs physio-chemical characteristics in order to understand which of them are more relevant for the antioxidant capacity of AOs.

In previous studies, we have modified the hydrophilic-lipophilic balance (HLB) of potent, natural antioxidants by grafting non-reactive alkyl chains of different length. The main purpose was to modify the partitioning of the antioxidants between the oil, interfacial and aqueous regions, to modify the effective interfacial concentrations and to analyze their effects on the AO efficiency, while maintaining their reactive moieties. Results showed that, regardless of the size of the droplets that make up the emulsion, the antioxidant efficiency for different series of homologous antioxidants bearing the same reactive moieties but of different hydrophobicity did not increase linearly with the hydrophobicity of compounds. In fact, a parabolic-like trend (cut-off effect) was observed, with the maximum activity found for those derivatives with alkyl chains of 4–12 carbon [5,6,7]. Other researchers reported similar results, [7,8,9] but they failed in correlating quantitatively the experimental results with the effective antioxidant concentrations. In all cases investigated, the exact chain length for maximum antioxidant efficiency depends on the nature of the polyphenolic moiety of the phenolipid [5,6,10,11,12,13,14,15] and on the composition of the emulsified system [5,16]. To date, most studies focused on investigating the relationships between the antioxidant efficiency and their distribution (and, in some cases, the effective interfacial concentration of antioxidants) for a number of series of homologous antioxidants [5,6,10,11,12,13,17]. However, the impact of the nature of their reactive moiety has not been sufficiently investigated in emulsions. In this work, we evaluated the antioxidant efficiency and the distribution of hydrophobic antioxidants (with bonded alkyl chains of 8 and 16 carbons) and their parent compounds with different reactive phenolic moieties in oil-in-water emulsions. We also determined some physiochemical and in vitro antioxidant properties including oxidation peak potential (*E*_pa_), DPPH radical scavenging activity (EC_50_) and reducing capacity (FRAP value) in an attempt to predict the relative antioxidant efficiency on the basis of the values of those parameters. For this purpose, caffeic acid (CA), 4-hydroxycinnamic acid (HCA), dihydrocaffeic acid (DCA), tyrosol (TY) and hydroxytyrosol (HT) and their phenolipids (Table 1) were selected for the study. The reactivity of phenolic antioxidants against peroxyl radicals depends, among others, on the number and position of phenolic hydroxyls-OH groups, the nature of the substituents on the aromatic ring and the presence of adjacent double bonds which allows a higher electron delocalization. Thus, the selected C8 and C16 antioxidants are quite hydrophobic but do have different reactivities. In attempting to correlate the observed antioxidant efficiencies with the interfacial concentrations and scavenging properties of compounds, Pearson’s correlation analysis was carried out considering the antioxidant capacity of the AOs, their first anodic peak potential, their reducing capacity (FRAP values), DPPH radical scavenging activity (EC_50_) and the effective concentration of the antioxidants in each region of the emulsified system. Moreover, a stepwise linear regression was employed to select the most likely predictors of the relative increase of emulsion stability and, thereby, to take a further step towards a deeper understanding of the main factors that affect the efficiency of antioxidants. This statistical analysis, though in this work was applied to a single emulsified system, should be considered as a first attempt in the search of a set of parameters that eventually will allow higher predictability in the search of the best antioxidant, or set of antioxidants, for emulsified systems.

## 2. Materials and Methods

### 2.1. Materials

All chemicals were of the highest purity available and used as received. Aqueous buffered solutions (citric acid/citrate; 0.04 M; pH 3.65) were employed in the preparation of emulsions. Olive oil-in-water emulsions were prepared by employing commercial olive oil stripped from their natural antioxidants. Briefly, natural tocopherols and phenols were stripped from commercial virgin olive oil by washing with 0.5 M NaOH solution and passing twice through an aluminum oxide column. Complete removal of tocopherols was confirmed by HPLC (Thermo Vanquish Horizon, Lisboa, Portugal) according to the IUPAC method 2.432. Removal of phenols was confirmed after SPE extraction by HPLC. Details can be found elsewhere [18]. Stripped oil was flushed with argon and stored in the dark at −20 °C to minimize its oxidation until it was used, within two weeks. To determine fatty acid composition, the methyl-esters were prepared according to the AOCS method Ch 2–91 (AOCS Press) and analyzed by GC (Perkin Elmer Clarus 480, Lisboa, Portugal). The oil composition was (given as percentage in weight): 11.6% of palmitic acid, 2.7% of stearic acid, 76.2% of oleic acid, 9.5% of linoleic acid and 0.5% of linolenic acid. Initial peroxide value and conjugated diene content (%) was 2.6 meq O_2_ and 0.075% respectively. The water employed in the emulsion preparation was of Milli-Q grade (conductivity < 0.1 mS cm^−1^). The citric acid and sodium citrate employed in the preparation of buffer solutions, Tween 20, tyrosol, hydroxytyrosol, 4-hydroxycinnamic acid, caffeic acid and dihydrocaffeic acid (all from Acros organics, Lisboa, Portugal) were of the highest purity and used as received. The chemical probe, 4-*N*-hexadecylbenzenediazonium tetrafluoroborate, 16-ArN_2_BF_4_, was prepared in high yield and purity from commercial 4-*N*-hexadecylaniline (Aldrich 97%) by diazotization following a published method [19].

### 2.2. Synthesis of Phenolipids

Several phenolipids with alkyl chain lengths of 8 (C8) and 16 carbons (C16) derived from tyrosol (TY8 and TY16), hydroxytyrosol (HT8 and HT16), 4-hydroxycinnamic acid (HCA8 and HCA16), caffeic acid (CA8 and CA16) and 3,4-dihydrocaffeic acid (DCA8 and DCA16) were synthetized. 

#### 2.2.1. Synthesis of Caffeates and Hydroxycinnamic Esters

Synthesis of alkyl caffeates and hydroxycinnamates of chain lengths of 8 and 16 carbons were prepared by using monomalonates and substituted benzaldehyde derivatives as starting material and applying the Verley-Doebner modification of the Knoevenagel condensation reaction as described by Costa et al. [6]. The compounds were identified, and their purity checked by ^1^H and ^13^C NMR. Results for CA8 and CA16 were in accordance with previously published results [6]. The results for HCA8 and HCA16 are provided in the Supplementary material S1.

#### 2.2.2. Synthesis of Tyrosol and Hydroxytyrosol Esters

The C8 derivatives were synthesized by direct esterification of HT and TY with the corresponding fatty acid and the C16 derivatives were prepared by transesterification with the corresponding fatty ethyl ester following the procedure described by Almeida et al [11]. The compounds were identified, and their purity checked by ^1^H and ^13^C NMR. Results for hydroxytyrosol octanoate (HT8) and hexadecanoate (HT16) were in accordance with previously published results and are not reproduced here [11]. The results for tyrosol octanoate (TY8) and hexadecanoate (TY16) are provided in the Supplementary material S2.

#### 2.2.3. Synthesis of Dihydrocaffeates

The DCA8 derivative was synthesized by direct esterification of dihydrocaffeic acid with the corresponding fatty alcohol, and the DCA16 derivative was prepared by transesterification with the corresponding hexadecyl acetate following the procedure described by Almeida et al [11]. The results for octyl (DCA8) and hexadecyl (DCA16) dihydrocaffeates are provided in the Supplementary material S3.

### 2.3. Preparation of Emulsions

Olive oil-in-water emulsions (4:6, O/W) were prepared from stripped olive oil, acidic water (0.04 M citrate buffer, pH 3.65) and using Tween 20 as emulsifier. The volume fraction of surfactant, Φ_I_, defined here after as Φ_I_ = V_surf_/V_emulsion_ was varied from Φ_I_ = 0.005 up to Φ_I_ = 0.04. Mixtures were stirred at high speed and room temperature for 1 min with the aid of a Polytronic PT-1600 homogenizer. 

### 2.4. Reactivity of the Different Phenols

#### 2.4.1. DPPH Radical Scavenging Efficiency 

The effects of the phenolipids alkyl chain on the free radical scavenging activity were investigated in bulk ethanolic solution by using the ability of phenolic compounds to reduce the DPPH radical at 25 °C [20]. The relative free antiradical activity was given by the EC_50_ value, defined as concentration of AO required to lower the initial DPPH^•^ concentration by 50% [6,10,11,20]. 

#### 2.4.2. Cyclic Voltammetry

A Hi-Tek potentiostat, type DT 2101, and a Hi-Tek wave generator type PPRl, connected to a Philips recorder, type PM 8043, and to a three electrode, home-built glass cell were used. Experiments were carried out using a glassy carbon working electrode and measured vs. a Ag/AgCl electrode in phosphate buffer solution at pH of 7.4 and at pH 3.65, a similar pH to that used in this work. 0.1 mL of a stock solution (10 mmol L^−1^ in ethanol) was diluted in 10 mL of aqueous buffer (KH_2_PO_4_/H_3_PO_4_ 0.1 mol L^−1^, pH 3.65, final AO concentration of 0.1 mmol L^−1^) in the electrochemical cell. Auxiliary experiments were carried out in the presence and absence of Tween 20 in order to check the effect of the emulsifier on the AOs *E*_pa_ values. The results displayed in Table 1 show that values for *E*_pa_ are not dependent on the length of the esters alkyl chain, in accordance with the results found when employing the DPPH^•^ assay and did not change significantly in the presence of Tween 20.

#### 2.4.3. Ferric-Reducing Antioxidant Potential (FRAP) Assay

The phenolic compounds ferric-reducing capacity was determined according to the procedure described by Benzie and Strain [21] with some modifications. Buffer solution (3 mL) was mixed with 0.1 mL of a freshly prepared ferric tripyridyltriazine (Fluka, Switzerland) solution, the FRAP reagent, and 0.1 mL of a methanolic phenol solution (1000 µM) at T = 37 °C. After 6 min of reaction, the absorbance at = 593 nm (Thermo Scientific Evoltution Array, Lisboa, Portugal) was determined against a blank. The reducing capacity was determined in a mixture (1:1) of acetate buffer 0.05 M, pH 3.65 and methanol. Standard solutions of Fe(II) in this mixture in the range of 100–2000 µmol/L (FeSO_4_^.^7H_2_O) were used for calibration. Changes in absorbance observed for each phenolic solution were converted into a FRAP value (in µM) by Equation (1):

FRAP value = (ΔA_593 nm_ test sample/ΔA_593 nrm_ standard) × Conc. Standard (μM) (1)

FRAP values were determined in quadruplicate.

### 2.5. Determining the Partition Constants and Distribution of Polyphenols in Intact Olive Oil-in-Water Emulsions: Application of the Pseudophase Kinetic Model

**Figure 1:**
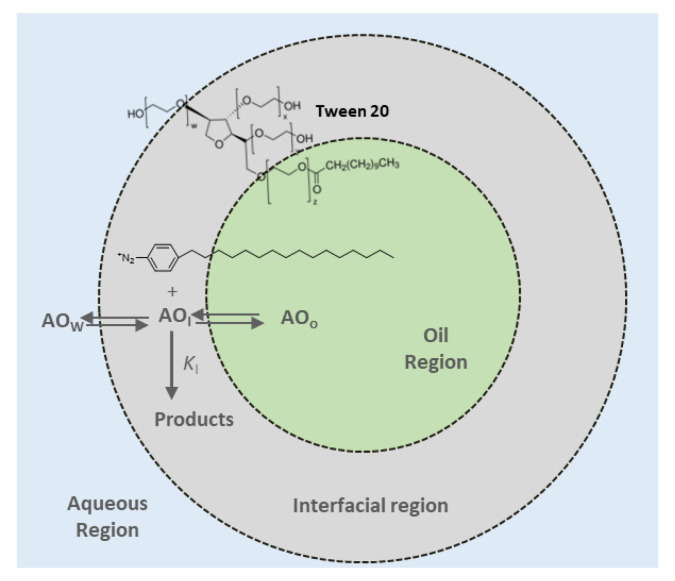
Partitioning of phenols between the different regions of a model emulsified system. *k*_I_ is the rate constant for the reaction between 16-ArN_2_^+^ and the AO in the interfacial region.

The phenolic compounds used in this study distribute in a different extent between the olive oil, interfacial, or aqueous regions of emulsions depending on their hydrophilic lipophilic balance (HLB) (Figure 1). Their distribution is defined by the partition constants between the olive oil–interfacial region, $P_{O}^{I}$, and aqueous–interfacial region, $P_{W}^{I}$, (Equations (2) and (3), subscripts O, I, and W indicate the oil, interfacial and aqueous regions, respectively), and the magnitudes between parentheses, indicate effective concentrations expressed in moles per liter of the region:

(2) $$P_{O}^{I}=({\mathrm{AO}}_{I})/({\mathrm{AO}}_{O})\;$$

(3) $$P_{W}^{I}=({\mathrm{AO}}_{I})/({\mathrm{AO}}_{W})$$

The phenolipids distribution was determined in the intact emulsions by employing a kinetic method that exploits the reaction between the compounds and the chemical probe 4-hexadecylbenzenediazonium (16-ArN_2_^+^) ion [22]. The probe is located at the emulsion’s interfacial region because 16-ArN_2_^+^ is at the same time an ionic surfactant and both oil and water insoluble (Figure 1). 

A derivatization method was used to determine the rate constant between the probe and the phenolic compound, as described in detail elsewhere [22]. Briefly, unreacted 16-ArN_2_^+^ ions react with a suitable coupling agent, the *N*-(1-naphthyl) ethylenediamine dihydrochloride (NED), producing a stable azo dye. The reaction mixture is then diluted with a 50:50 (*v*:*v*) BuOH:EtOH solution and the absorbance determined spectrophotometrically at λ = 572 nm. In order to have reliable rate constant results, experimental conditions were optimized, so that the reaction of 16-ArN_2_^+^ with NED (t_1/2_ < 10 s) was much faster than the reaction of 16-ArN_2_^+^ with AOs (t_1/2_ > 30 min) [22]. Reactions were carried out under pseudo-first order conditions ([AO] >>> [16-ArN_2_^+^]) and followed for at least 2–3 t_1/2_. *k*_obs_ Values were obtained by fitting the absorbance-time pairs of data to the integrated first order (Equation (S2)), using a non-linear least squares method provided by GraFit 5.0.5.

### 2.6. Antioxidant Efficiency of Phenolipids in Emulsions

The efficiency of phenolipids and of the parent antioxidants in olive oil emulsions was determined, as in previous works, [23,24,25] by employing the Schaal Oven Test performed at T = 60 °C. 4:6 Olive oil-in-water emulsions were prepared as described above, in the presence of AO (final AO concentration of 0.24 mmol L^−1^ in total volume of the emulsion). Emulsions without AO were used as control. Every 12 hours, samples were vortexed for 1 min to minimize creaming. The degree of oxidation in the emulsions over time was evaluated by monitoring formation of conjugated dienes (CD) according to the AOCS Official Method Ti 1a 64. After being vortexed, a 50 µL aliquot of the emulsion was diluted to 10 mL with ethanol and the absorbance measured at 233 nm. 

In order to evaluate the antioxidant efficiency of compounds, the relative increase in the oxidative stability was determined by employing Equation (4):

Relative increase in the oxidative stability = (t_(AO)_ − t_(C)_)/t_(C)_ (4)

where t_(AO)_ and t_(C)_ are the time necessary for the samples containing each AO or the control respectively, to increase by 0.5% their conjugated diene content.

### 2.7. Statistical Analysis.

Kinetic experiments between the chemical probe 16-ArN2+ and the AO were run in triplicate for 2–3 t_1/2_. The observed rate constant (*k*_obs_) values were within ±7–9% with typical correlation coefficients over 0.995. Cyclic voltametric determinations and oxidation experiments were run at least in triplicate. Statistical analyses were performed using SPSS software (IBM SPSS Statistics for Windows, Version 26.0. Armonk, NY: IBM Corp). The comparison of the means was performed by a one-way analysis of variance (ANOVA, with Tukey’s HSD multiple comparison) with the level of significance set at *p* < 0.05. 

Correlation coefficients were calculated using Pearson product moment correlation. Stepwise linear regression analysis (SLRA) was used to evaluate how much variability in increase of relative stability could be explained by each independent predictor. In SLRA calculations, only compounds with distribution data were included. A probability-of-F-to-enter < 0.150 and probability-of-F-to-remove >0.200 was used to select the predictors in the regression model.

## 3. Results and Discussion

### 3.1. Reactivity of Phenolic Compounds 

In order to evaluate the radical-scavenging ability of phenolic compounds some antioxidant capacity assays, namely, the DPPH^•^ assay, are often used [26]. The DPPH^•^ method has the advantage of being simple and can be used to establish a rank of the radical scavenging activity without the interference of several factors, such as distribution/partitioning properties and metal chelation [27,28]. 

Table 1 shows similar EC_50_ values for the AOs within each series, indicating a negligible effect of the length of the alkyl chain grafted to the polyphenolic moiety on their reactivity with DPPH^•^. On the other hand, when comparing the reactivity between series, Table 1 also shows that the radical scavenging activity of AOs depends on their molecular structure.

Compounds with only one -OH group in the aromatic ring showed no significant reactivity against the DPPH radical, in agreement with literature reports [29]. In contrast, catecholic compounds exhibited high free radical scavenging activity against DPPH^•^, with dihydrocaffeates being better scavengers than caffeates and better than those derived from hydroxytyrosol. It is interesting to note that the conjugation found in caffeic acid and caffeic acid phenolipids actually decrease the reactivity of these compounds against the DPPH radical when compared with dihydrocaffeic acid and its phenolipids, where this conjugation is absent. This different reactivity is more pronounced between the parental compounds, with the dihydrocaffeic acid showing the best antiradical capacity of all compounds.

The in vitro antioxidant capacity of phenols has been established, and generally accepted to be closely related to their redox properties and electron transfer reactions and correlation between redox properties, and the antioxidant activity of low molecular weight antioxidants has been reported [30]. Mechanisms of the inhibitory effect of phenolic compounds on lipid oxidation have been extensively studied in bulk solution and some surfactant-based systems [31,32,33]. Several mechanisms that may operate simultaneously have been proposed: (i) hydrogen–atom transfer and/or proton–coupled electron transfer (HT); (ii) sequential electron transfer–proton transfer (SETPT); (iii) sequential proton loss–electron transfer (SPLET) and (iv) adduct formation [34,35]. Whatever is the predominant mechanism, it results in the production of a phenoxy radical. It is currently accepted that the electrochemical oxidation occurring at the –OH groups, is largely influenced by the number and position of the substituents linked to the aromatic ring [36]. In addition, environmental parameters such as the acidity also affect the phenols’ antiradical scavenging activity, redox behavior and oxidation product formation [37]. It is not strange, therefore, that electrochemical techniques such as cyclic voltammetry have been used as an important tool to predict the antioxidant capacity of molecules, since compounds that have lower oxidation potentials, and therefore are more easily oxidized, usually show better antioxidant activities.

In this work, we determined by cyclic voltammetry the oxidation peak potentials for all compounds in bulk solution at pH 3.65 and 7.4 (Table 1). We also studied the influence of the presence of tween 20 micelles in the solution.

Within each phenolipid series, no significant difference (*p* < 0.05) in the values for the first anodic potential was detected either in the presence or absence of surfactant. The introduction of an alkylamide or an alkylester group in the side chain of the aromatic ring of the AOs does not lead to significant changes in the redox potentials of simple catecholic compounds [38,39]. However, with the monophenols, there was a significant decrease in the anodic potential value at pH 7.4 when TY and HCA were esterified probably due to a more suppressive influence of the carboxylate group (–COO^−^) on the deprotonation of the aromatic hydroxyl group [38]. Because of its electron-donating effects, –COO^−^ increases the proton affinity, which decreases their redox potentials, where the deprotonation of ArOH (phenolic compound) is supported by the electron-withdrawing effect of the ester moiety (–COOR). This is in accordance with the results found by Foti et al. [40]. Important differences on the first anodic potential were found between the different phenolipid series. The phenolic –OH undergoes anodic oxidation according to the stability of the phenoxy radical formed which is dependent on the substituents in the aromatic ring. The values of the first anodic potential for the families with only one hydroxyl group on the aromatic ring (tyrosol and hydroxycinnamic acid) were much higher than the ones containing catecholic moieties (hydroxytyrosol, caffeic acid and dihydrocaffeic acid) in accordance with previous observations for caffeic acid and *p*-coumaric acid [29]. Mono-phenols have been described as oxidizing in a one-electron, one-proton irreversible step, to a phenoxy radical [32]. On the other hand, the oxidation mechanism of AOs bearing the catecholic moiety involves a two-electron and two-proton reversible process [36] that appears at less positive potentials due to the higher stability of the radical formed, when compared to that of the mono-phenol. In the case of esters of dihydrocaffeic acid, *E*_pa_ values have a significantly lower valor than those of caffeic acid, suggesting that the extension of the resonance over the alkyl side chain removes electronic density at the aromatic hydroxyl groups, decreasing their ability to donate H-atoms. These observations are in line with the bond dissociation enthalpy (BDE) and the ionization potential (IP) values calculated for caffeic and dihydrocaffeic acid that were found to be lower for the former [41]. Molecular descriptors, such as the BDE and IP values are used to estimate the H-atom- and electron-donating ability of an AO, respectively. Therefore, the absence of a double bond in the side chain, as seen in the dihydrocaffeic series, leads to lower redox potential values and an increase in the radical scavenging activity in accordance with the results obtained in DPPH radical scavenging assay. 

At pH 3.65, although there is small difference between the *E*_pa_ values for the catechol series, the overall rank was DCA < CA < HT << Ty = HCA (*p* < 0.05). Since the redox process involves the participation of protons, at higher pH, the electron loss is easier and therefore, at pH 7.4, all compounds showed a much lower first anodic potential, with the differences being now significant between the catecholic series, with the rank DCA < HT < CA << HCA = Ty (*p* < 0.05). 

In the presence of transition metal ions, both radical scavenging and metal chelation properties contribute to the antioxidative capacity of phenols. However, phenols may chelate transition metal ions hence reducing metal-induced oxidative reactions. They also have a high capacity to reduce metallic ions producing reduced metal species with a higher pro-oxidant capacity. In fact, oxidized metals form many complexes with phenolic compounds but a transfer of an electron from the ligand to the metal can occur, depending on the stability of the chelate. Therefore, reducing properties of polyphenols are of high relevance for their antioxidant capacity [42,43,44]. Since a strong chelating buffer solution was used in the preparation of emulsions, the reduction of metallic species is not expected in this work. However, in most cases, the inhibition of the oxidative radical chain involves a mechanism where a redox reaction takes place as already discussed. Therefore, the determination of the iron-reducing ability of phenols, has been used frequently as a measure of their potential antioxidant capacity. Therefore, the ferric-reducing ability of phenolic compounds was evaluated by the FRAP assay (Table 1).

Phenolic compounds were evaluated by the FRAP assay at pH 3.65 (Table 1). As expected, the reducing capacity of compounds with only one aromatic hydroxyl group was much lower than the reducing capacity of catechols (Table 1). Nevertheless, catecholic parent compounds showed some differences between their reducing capacity, with the DCA showing the best reducing capacity followed by CA, and with HT showing a lower reducing capacity (Table 1). These results are in accordance with the rank found for their first anodic potential values (Table 1), as lower values usually mean easier electron transfer reactions.

In general, it was also observed that the reducing capacity of parental phenolic compounds was higher than the reducing capacity of their esters and this capacity decreased with the increase in alkyl length of the phenolipid (Table 1). It is known that the stability of complexes formed between ligands and metals contributes to the lower reducing capacity of the former compounds [43,44]. However, in this case, once the complex is formed, the same reactivity and stability of the complex formed within each series of phenolipids is expected, as the stability of the complex is influenced by the same groups attached to the aromatic ring. Thus, the different reducing power observed between the C8 and the C16 derivatives of each series must be caused by the steric hindrance associated with the longer acyl/alkyl side chain length that prevents the initial formation of the complex. These results are in accordance with the results found for a series of nitrohydroxytyrosyl esters, where the esters also showed a decrease in their FRAP values with the length of the alkyl chain (from C2 up to C16) [45].

### 3.2. Determining the Partition Constants and Distribution of Polyphenols in Intact Olive Oil-in-Water Emulsions: Application of the Pseudophase Kinetic Model

The partition constants and distribution of DCA and HCA series in intact olive oil-in-water emulsions were determined by the pseudophase kinetic model [22]. As in our previous papers, *k*_obs_ values were determined by employing a derivatization method (Supplementary material, S4). Figure S1 (Supplementary material) shows a typical example of the kinetics obtained by the reaction of the probe with the derivative octyl dihydrocaffeate (DCA8). The observed rate constant values, *k*_obs_, for the reaction between the AOs and 16-ArN_2_^+^ were measured in 4:6 (O/W) emulsions formed by olive oil, citrate buffer (0.04 M, pH = 3.65), and Tween 20 (emulsifier) at different volume fractions (Φ_I_ = 0.005–0.04). *k*_obs_ values were within ± 7–9 % with typical correlation coefficients over 0.995. An illustrative example is shown in Figure S2, Supplementary material. 

Partition constant values for the distribution of caffeic acid, 4-hydroxycinnamic acid dihydrocaffeic acid and hydroxytyrosol derivatives are displayed in Table 1. Values for CA and HT are in keeping with reported literature values [6,11]. For tyrosol, its esters and for 4-hydroxynamic acid, it was not possible to determine the observed constant, *k*_obs_, since the reactivity of these compounds with the arenodiazonium ions was very low. Distribution of dihydrocaffeic acid and its esters was determined in the present work.

**Figure 2:**
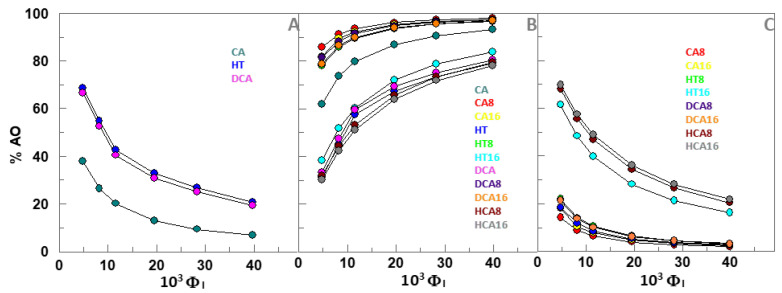
Percentage of the different AOs in the aqueous (**A**), in the interfacial (**B**) and oil (**C**) regions as a function of the Tween 20 emulsifier fraction (Φ_I_), for a 4:6 (O/W) emulsion (olive oil/citrate buffer, pH = 3.65/Tween 20).

Knowing the partition constants values $P_{O}^{I}$ and $P_{w}^{I}$ (Table 1), the percentage of antioxidant in the oil, aqueous and interfacial regions of the emulsions was determined for each value of Φ_I_ by applying Equations (S5) and (S6) of the simplified pseudophase model (Supplementary material) (Figure 2). Results show that AOs with 8 and 16 carbon alkyl chains were mostly present in the interfacial region of the emulsified system (%AO_I_ > 70%). In all series, the highest percentage is found for the C8 derivative (Figure 2) with the highest percentage found for the octyl caffeate (about 90%). 

**Figure 3:**
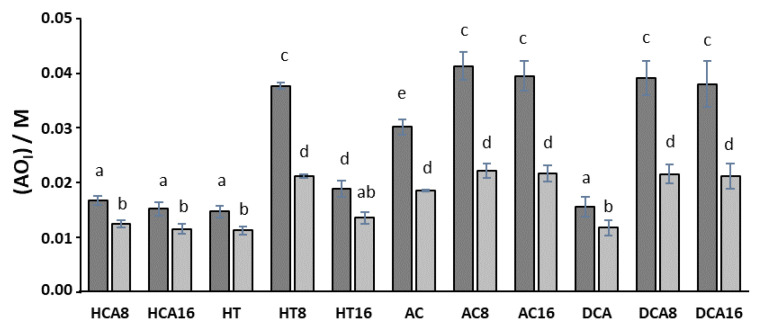
Effective concentration of AO in the interfacial region of 4:6 (O/W) emulsions (olive oil/citrate buffer, 0.04M, pH = 3.65/Tween 20) for the emulsifier fractions of Φ_I_ = 0.005 (dark grey color) and Φ_I_ = 0.01 (light gray color), [AO_T_] = 0.24 mM. Different letters (superscripts) indicate samples that were significantly different (*p* < 0.05).

Results also show that the percentage of AO_I_ does not correlate directly with the length of the alkyl chain, that is, with the hydrophobicity of AO, since, in all cases, the 8-carbon esters are present in a higher percentage in the interfacial region than the compounds with 16 alkyl carbons (Figure S3, Supplementary material). Keeping with previous results (5–10), for any AO, the percentage of AO_I_ increases upon increasing Φ_I_ (Figure 2B). However, this increases in the percentage of AO_I_, does not compensate the dilution that occurs due to the increase in the interfacial volume and, therefore, the effective concentration of AOs decreases upon increasing Φ_I_ (Figure 3).

### 3.3. Antioxidant Activity of Phenolic Compounds in Olive Oil-in-Water Emulsions

**Figure 4:**
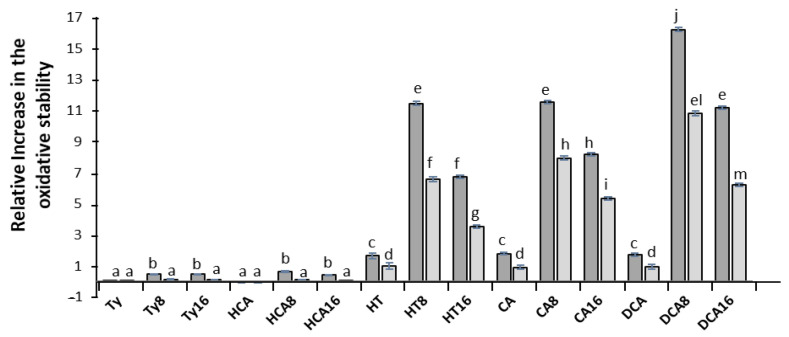
Relative increase in the oxidative stability of 4:6 (O/W) emulsions (olive oil/citrate buffer, 0.04 M, pH = 3.65/Tween 20) containing the antioxidants prepared with emulsifier fractions of Φ_I_ = 0.005 (dark grey color) and Φ_I_ = 0.01 (light gray color) Mean (error represent the standard deviation) of triplicate stored samples ([AO_T_] = 0.24 mM, T = 60 °C). Superscripts indicate samples that were significantly different (*p* < 0.05).

The antioxidant activity of AOs in intact emulsions was evaluated as in previous works by employing the Schaal oven test at T = 60 °C [5,6,10,11,12,46] and followed by the determination of the sample conjugated diene content (Figure S4, Supplementary data). We have shown in previous work [6,11,12,17,23] that the increase in the CD content is positively correlated (r^2^ > 0.970) with the increase in the peroxide value (PV) (AOCS Official Method Cd 8-53). The relative increase in the oxidative stability of emulsions in the presence of AOs can be observed in Figure 4. At Φ_I_ = 0.005, the order of antioxidant efficiency (*p* < 0.05) was: DCA8 > CA8 = HT8 = DCA16 > CA16 > HT16 > HT = CA = DCA > HCA8 = TY8 = TY16 = HCA16 > TY = HCA = Control. At Φ_I_ = 0.01, the order of antioxidant effectiveness was very similar to that obtained above for 0.5% emulsifier: DCA8 > CA8 > HT8 > DCA16 > CA16 > HT16 > HT = CA = DCA > TY8 = TY16 = HCA8 = HCA16 = TY = HCA = Control.

Phenolipids containing only one aromatic -OH group were only efficient in emulsions with low surfactant volume fractions. Their efficiency, although small, was superior to the activity of parent compounds, which did not exhibit any antioxidant efficiency at all (Figure 4), in accordance with their radical scavenging activity and redox properties. Most probably, the reason of the increase in their efficiency in emulsions is a consequence of their accumulation at the interface, increasing their effective concentration, and therefore, the rate of the inhibition reaction.

Phenolipids containing catecholic moieties were much more efficient than the monophenolic derivatives (in keeping with their high radical scavenging activity and redox properties), increasing the stability of emulsions from ~4-up to 16-fold depending on the AO and the Φ_I_ used in the preparation of the emulsion. The antioxidant activity shown by the parent compounds (Figure 4) was smaller than that observed for phenolipids due to their lower concentration at the interfacial region. For all phenolipid series, emulsions containing the C8 derivative were more stable (~1.5 fold) than emulsions containing the C16 derivative. Thus, the antioxidants that are found in greater percentages at the interfacial region, for the same amount of emulsifier, show a better antioxidant efficiency. 

Results also show that the antioxidant efficiency for all compounds decreases with the increase in the volume fraction from Φ_I_ = 0.005 to Φ_I_ = 0.01 (Figure 4). The concentration of AO in the interfacial region is about 63-180 times higher than the stoichiometric concentration of AO in the emulsion ([AO_T_] = 0.24 mM) at Φ_I_ = 0.005 but only 47–93 times higher at Φ_I_ = 0.01. Thus, interfacial concentration decreases upon increasing Φ_I_ in spite of the percentage of antioxidant in the interfacial region increases (Figure 2). We must bear in mind that an increase in Φ_I_ leads to an increase in the interfacial volume resulting in a dilution of the antioxidant in the interfacial region (Figure 3). These results confirm that the concentration of an antioxidant at the interfacial region is a major factor that controls the antioxidant efficiency. 

However, DCA8 showed to be a better AO than CA8 and HT8 in spite of lower or similar concentration at the interfacial region. The same was observed for DCA16, showing a better antioxidant efficiency then CA16. Therefore, DCA8 and DCA16 efficiency should be lower as we demonstrated that there is a correlation between antioxidant efficiency and the concentration of AO in the interfacial region. These apparently contradictory results can be rationalized in terms of the lower reduction potential shown by dihydrocaffeates (*E*_pa_ = 0.339 V for DCA8) when compared with the caffeic acid (*E*_pa_ = 0.379 V for CA8) and hydroxytyrosol series (*E*_pa_ = 0.419 V for HT8). Despite a small difference in the interfacial concentration of CA8 and DCA8 when compared with the interfacial concentration of CA16 and DCA16, a significant difference in their oxidative stability was observed, with the C8 derivatives being much more active. As observed in the interactions with the iron ion, a steric effect given by the longer chain of C16 derivatives may hinder the inhibition reaction of the AO with a bulky triacylglycerol radical, causing a decrease in their antioxidant capacity. Thus, despite the slightly higher concentration in the interface shown by the DCA8 (~39 mM) when compared to DCA16 (~38 mM), octyl dihydrocaffeate showed to be the most efficient antioxidant of all phenolipids tested, with a much higher antioxidant efficiency than the DCA16 (~16-fold and ~11-fold increase in the oxidative stability of the sample prepared with Φ_I_ = 0.005 and containing DCA8 and DCA16, respectively, in relation to the control).

### 3.4. Pearson Correlation and Stepwise Linear Regression Analysis (SLRA)

Prediction of the antioxidant efficiencies of antioxidants is of general importance in food chemistry and of interest for the food industry. Therefore, Pearson correlation analysis was performed by considering the first anodic peak potential; EC_50_ values (5, 60 min); antioxidant concentration in each region of the system; emulsifier fraction, FRAP value and the relative antioxidant efficiency in emulsions 4:6 (olive oil/citrate buffer, 0.04 M, pH = 3.65/Tween 20), (Table 2). 

The factors that show the highest correlation with the relative increase in the oxidative stability of emulsions are the effective concentration of antioxidant at the interfacial region (ρ = 0.820), followed by the anodic potential value (ρ = −0.677) and, to a lesser extent, the radical scavenging activity (ρ = −0.654) (Table 2). The results confirm the importance of determining the effective concentration of AOs in the interfacial region in addition to the H-donating ability of the antioxidants (*E*_pa_ values) in predicting the antioxidant efficiency of compounds in emulsions. 

Stepwise linear regression analysis (SLRA) was applied to the data to select the predictors that better explain the increase in emulsion oxidative stability by the action of AOs. To build the model, F < 0.15 was used as the variable to enter and probably of F > 0.2 as the variable to remove. Three models were built (Table 3).

The concentration of AO in the interfacial region, (AO_I_), explains about 67 % of the variance in the data (model 1) and, together with the *E*_pa_ (model 2), it can explain about 72%, confirming the importance of these predictors for the antioxidant capacity of molecules. Besides the lower contribution of (AO_W_), a further model including this variable could be obtained (model 3, Table 3) where almost 82% of the results could be explained. The coefficients of the model obtained are all significant (<0.05) and the variance inflation factors (VIF) show that multicollinearity in the model is negligible. (Supplementary Material, Table S1).

Analyzing the values of the standardized coefficients (Supplementary Material, Table S1) we obtain information on the factors that most influence oxidative stability. The concentration of the AO at the interface (0.496) appears to be the most important predictor as stability increases with this variable. This relationship has been reported in several studies [5,6,10,11,12]. *E*_pa_ (−0.425) is the second most important variable and in this case oxidative stability decreases as *E*_pa_ increases. Finally, the concentration in water (−0.392) indicates that the oxidative stability of samples is also quite affected by this factor and decreases when this concentration increases.

Prediction of the best antioxidant, or set of antioxidants, for inhibiting the oxidation of a particular emulsified system is still far from being practical for routine usage. Part of the problem lies in the absence of sets of molecular parameters or system properties that, eventually, allow a trustable prediction. Antioxidant properties work cooperatively in emulsified (and other) systems and it is important to be able to translate individual values of antioxidant properties into those of global antioxidant efficiency. Thus, models to quantify the relative contributions of individual properties are of interest and studies involving a wider range of antioxidants and emulsified systems are currently being analyzed and will be part of future reports. 

## 4. Conclusions

All catecholic phenolipids, and in particular the C8 derivatives, have been proven to be suitable antioxidants for the protection of olive oil-in-water emulsions from oxidation by air. Nevertheless, the antioxidant efficiency of dihydrocaffeic acid derivatives in these systems stands out, with the ability to increase the oxidative stability of emulsions more than 16 times in relation to the control. 

Pearson´s correlation between the oxidative stability of emulsions and (AO_I_) (Figure 4) has the highest value (0.820), confirming that, all other things being equal, there is a direct correlation between the interfacial concentration of AOs and their efficiency in inhibiting lipid oxidation in emulsions, highlighting the importance of determining the effective concentration of AOs in the interfacial region. This molecular parameter can, thus, be safely employed in predicting the antioxidant efficiency of the compounds in the emulsified systems as shown in this and in previous papers. 

Stepwise Linear Regression Analysis showed that (AO_I_) could explain most of the variance observed (67.2%) and together with *E*_pa_ and (AO_W_), could explain almost 82% of the variance in the relative oxidative stability of samples containing AOs. These sets of results from SLRA not only identify (or confirms) the most important predictors for the oxidative stability of the samples studied but also allows to obtain quantitative estimates of the effects of these predictors. Understanding the relative contribution of all these factors to the overall antioxidant efficiency of antioxidants will, hopefully, permit in the near future the development of guidelines, based on measurable parameters, for selecting the most efficient AO for a particular application. This may enable the successful design of alternative, effective antioxidants that can be eventually employed to minimize the spoilage of foods by oxygen.

## Figures and Tables

**Table 1:** DPPH% Radical-scavenging capacity, EC_50_^a^, oxidation peak potentials, *E*_pa_, FRAP values and values for $P_{w}^{I}$ and $P_{O}^{I}$ of compounds in O/W emulsions.

			*E*_pa_ (V vs. Ag/AgCl)			EC_50_^a^ (mol AO/mol DPPH)			Emulsion	
Compound		R	pH 7.4	pH 3.65	pH 3.65 + Tween 20	5 min	60 min	FRAP Value (μM)	$P_{W}^{I}$	$P_{O}^{I}$
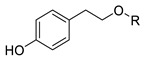	**TY**	H	0.645 ± 0.010 ^a^	0.798 ± 0.015 ^a^	-	21 ± 2 ^a^	20 ± 2 ^a^	54.7 ± 2.5 ^a^	-	-
	**TY8**	-CO-(CH_2_)_6_CH_3_	0.561 ± 0.008 ^b^	0.763 ± 0.005 ^a^	-	23 ± 2 ^a^	23 ± 3 ^a^	19.6 ± 3.6 ^b^	-	-
	**TY16**	-CO-(CH_2_)_14_CH_3_	0.583 ± 0.004 ^b^	0.783 ± 0.014 ^a^	-	23 ± 4 ^a^	23 ± 3 ^a^	12.5 ± 1.8 ^c^	-	-
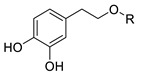	**HT**	H	0.217 ± 0.002 ^c^	0.411 ± 0.011 ^b^	0.410 ± 0.010 ^a^	0.323 ± 0.005 ^b^	0.258 ± 0.004 ^b^	1523 ± 19 ^d^	53 ± 7	-
	**HT8**	-CO-(CH_2_)_6_CH_3_	0.235 ± 0.003 ^c^	0.419 ± 0.006 ^b^	0.392 ± 0.009 ^a^	0.295 ± 0.005 ^b^	0.243 ± 0.003 ^b^	1193 ± 10 ^e^	-	296 ± 85
	**HT16**	-CO-(CH_2_)_14_CH_3_	0.222 ± 0.002 ^c^	0.403 ± 0.020 ^b^	0.391 ± 0.010 ^a^	0.331 ± 0.011 ^b^	0.268 ± 0.007 ^b^	653 ± 4 ^f^	-	52 ± 5
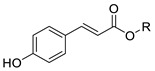	**HCA**	H	0.612 ± 0.006 ^d^	0.805 ± 0.005 ^c^	-	23 ± 2 ^a^	21 ± 2 ^a^	59.9 ± 4.2 ^a^	-	-
	**HCA8**	-(CH_2_)_7_CH_3_	0.579 ± 0.003 ^e^	0.760 ± 0.003 ^d^	-	24 ± 2 ^a^	22 ± 3 ^a^	56.4 ± 1.7 ^a^	-	39 ± 2
	**HCA16**	-(CH_2_)_15_CH_3_	0.566 ± 0.010 ^e^	0.787 ± 0.018 ^d^	-	24 ± 3 ^a^	23 ± 3 ^a^	54.1 ± 1.7 ^a^	-	36 ± 3
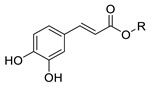	**CA**	H	0.265 ± 0.001 ^f^	0.394 ± 0.018 ^e^	0.411 ± 0.009 ^a^	0.330 ± 0.005 ^b^	0.344 ± 0.005 ^c^	1765 ± 12 ^g^	204 ± 16	-
	**CA8**	-(CH_2_)_7_CH_3_	0.263 ± 0.003 ^f^	0.379 ± 0.008 ^e^	0.387 ± 0.010 ^a^	0.293 ± 0.012 ^b^	0.199 ± 0.005 ^d^	1617 ± 24 ^h^		502 ± 32
	**CA16**	-(CH_2_)_15_CH_3_	0.260 ± 0.001 ^f^	0.378 ± 0.018 ^e^	0.386 ± 0.008 ^a^	0.317 ± 0.003 ^b^	0.196 ± 0.002 ^d^	1078 ± 15 ^i^		376 ± 35
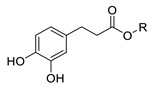	**DCA**	H	0.157 ± 0.004 ^g^	0.344 ± 0.007 ^f^	-	0.204 ± 0.003 ^c^	0.149 ± 0.006 ^e^	2921 ± 59 ^j^	58 ± 13	-
	**DCA8**	-(CH_2_)_7_CH_3_	0.155 ± 0.003 ^g^	0.339 ± 0.017 ^f^	-	0.266 ± 0.005 ^d^	0.267 ± 0.008 ^b^	1613 ± 24 ^h^	-	368 ± 29
	**DCA16**	-(CH_2_)_15_CH_3_	0.150 ± 0.008 ^g^	0.349 ± 0.026 ^f^	-	0.268 ± 0.006 ^d^	0.270 ± 0.004 ^b^	1513 ± 21 ^d^	-	220 ± 54

^a^ The antiradical activity was defined as the relative concentration of antioxidant required to lower the initial DPPH% concentration by 50% (moles of AO/moles of DPPH^•^) obtained at different reaction times, T = 25 °C. Superscripts in the same column indicate means that were significantly different (*p* < 0.05).

**Table 2:** Pearson correlation between different factors and the emulsions’ oxidative stability.

	(AO_I_)	(AO_w_)	(AO_O_)	*E*_pa_	EC_50_		FRAP
				pH = 3.65	5 min	60 min	
Oxidative Stability	0.820 **	−0.470 *	−0.184	−0.677 **	−0.654 *	−0.654 *	−0.434 *

* Correlation is significant at the 0.05 level. ** Correlation is significant at the 0.01 level (two tailed).

**Table 3:** Cumulative (Cum) R^2^, adjusted (Adj.) R^2^ and partial (Part) R^2^ in the stepwise linear regression analysis.

Model	Predictors	R	Cum.R^2^	Part.R^2^
1	(AO_I_)	0.820	0.672	0.672
2	(AO_I_) *E*_pa_	0.847	0.717	0.044
3	(AO_I_) *E*_pa_ (AOw)	0.905	0.819	0.103

## Supplementary Materials

The following are available online at https://www.mdpi.com/article/10.3390/foods10051028/s1, ^1^H and ^13^C NMR chemical shifts of synthetized compounds. Figure S1: Illustrative variations of absorbance of azo dye from the reaction between chemical probe and NED with time in olive oil-in-water emulsions containing DCA8. Figure S2: Representation of *k*_obs_ and 1/*k*_obs_ vs. a fraction of emulsifier (Φ_I_). Figure S3. Percentage of AO in the interface region of emulsions 4:6 (oil/citrate buffer, 0.04 M, pH = 3.65/ Tween 20) as a function of the number of carbon atoms in the alkyl chain, for each family, for the fraction of emulsifier, Φ_I_ = 0.005. Figure S4. Effects of the AO on the lipid oxidation reaction kinetic of 4:6 (O/W) emulsions (olive oil/citrate buffer, 0.04 M, pH = 3.65/Tween 20) prepared with emulsifier fractions of Φ_I_ = 0.01 (A) and 0.005 (B). Mean of triplicate stored samples ([AO_T_] = 0.24 mM, T = 60 °C). Table S1. Stepwise linear regression analysis (SLRA) coefficients.

## References

[1] McClements D.J., Decker E.A., Weiss J. (2007). Emulsion-Based Delivery Systems for Lipophilic Bioactive Components. J. Food Sci..

[2] Frankel E.N. (2014). Lipid Oxidation.

[3] Waraho T., McClements D.J., Decker E.A. (2011). Mechanisms of lipid oxidation in food dispersions. Trends Food Sci. Technol..

[4] Akoh C.C. (2017). Food Lipids: Chemistry, Nutrition and Biotechnology.

[5] Ferreira I., Costa M., Losada-Barreiro S., Paiva-Martins F., Bravo-Díaz C. (2018). Modulating the interfacial concentration of gallates to improve the oxidative stability of fish oil-in-water emulsions. Food Res. Int..

[6] Costa M., Losada-Barreiro S., Paiva-Martins F., Bravo-Díaz C., Romsted L.S. (2015). A direct correlation between the antioxidant efficiencies of caffeic acid and its alkyl esters and their concentrations in the interfacial region of olive oil emulsions. the pseudophase model interpretation of the “cut-off” effect. Food Chem..

[7] Laguerre M., López Giraldo L.J., Lecomte J., Figueroa-Espinoza M.-C., Baréa B., Weiss J., Decker E.A., Villeneuve P. (2009). Chain Length Affects Antioxidant Properties of Chlorogenate Esters in Emulsion: The Cutoff Theory Behind the Polar Paradox. J. Agric. Food Chem..

[8] Sørensen A.-D.M., Durand E., Laguerre M., Bayrasy C., Lecomte J., Villeneuve P., Jacobsen C. (2014). Antioxidant Properties and Efficacies of Synthesized Alkyl Caffeates, Ferulates, and Coumarates. J. Agric. Food Chem..

[9] Laguerre M., López Giraldo L.J., Lecomte J., Figueroa-Espinoza M.-C., Baréa B., Weiss J., Decker E.A., Villeneuve P. (2010). Relationship between Hydrophobicity and Antioxidant Ability of “Phenolipids” in Emulsion: A Parabolic Effect of the Chain Length of Rosmarinate Esters. J. Agric. Food Chem..

[10] Costa M., Losada-Barreiro S., Paiva-Martins F., Bravo-Díaz C. (2017). Physical evidence that the variations in the efficiency of homologous series of antioxidants in emulsions are a result of differences in their distribution. J. Sci. Food Agric..

[11] Almeida J., Losada-Barreiro S., Costa M., Paiva-Martins F., Bravo-Díaz C., Romsted L.S. (2016). Interfacial Concentrations of Hydroxytyrosol and Its Lipophilic Esters in Intact Olive Oil-in-Water Emulsions: Effects of Antioxidant Hydrophobicity, Surfactant Concentration, and the Oil-to-Water Ratio on the Oxidative Stability of the Emulsions. J. Agric. Food Chem..

[12] Meireles M., Losada-Barreiro S., Costa M., Paiva-Martins F., Bravo-Díaz C., Monteiro L.S. (2019). Control of antioxidant efficiency of chlorogenates in emulsions: Modulation of antioxidant interfacial concentrations. J. Sci. Food Agric..

[13] Silva R., Losada-Barreiro S., Paiva-Martins F., Bravo-Díaz C. (2017). Partitioning and antioxidative effect of protocatechuates in soybean oil emulsions: Relevance of emulsifier concentration. Eur. J. Lipid Sci. Technol..

[14] Losada-Barreiro S., Costa M., Bravo-Díaz C., Paiva-Martins F. (2014). Distribution and antioxidant efficiency of resveratrol in stripped corn oil emulsions. Antioxidants.

[15] Losada-Barreiro S., Bravo-Díaz C., Costa M., Paiva-Martins F. (2014). Distribution of catechol in emulsions. J. Phys. Org. Chem..

[16] Losada-Barreiro S., Bravo Díaz C., Paiva Martins F., Romsted L.S. (2013). Maxima in antioxidant distributions and efficiencies with increasing hydrophobicity of gallic acid and its alkyl esters. The pseudophase model interpretation of the ″Cut-off effect. J. Agric. Food Chem..

[17] Costa M., Losada-Barreiro S., Bravo-Díaz C., Vicente A.A., Monteiro L.S., Paiva-Martins F. (2020). Influence of AO chain length, droplet size and oil to water ratio on the distribution and on the activity of gallates in fish oil-in-water emulsified systems: Emulsion and nanoemulsion comparison. Food Chem..

[18] Lisete-Torres P., Losada-Barreiro S., Albuquerque H., Sánchez-Paz V., Paiva-Martins F., Bravo-Díaz C. (2012). Distribution of Hydroxytyrosol and Hydroxytyrosol Acetate in Olive Oil Emulsions and Their Antioxidant Efficiency. J. Agric. Food Chem..

[19] Gunaseelan K., Romsted L.S., Gallego M.J.P., Gonzalez-Romero E., Bravo-Diaz C. (2006). Determining α-Tocopherol Distributions between the Oil, Water, and Interfacial Regions of Macroemulsions: Novel Applications of Electroanalytical Chemistry and the Pseudophase Kinetic Model. Adv. Colloid Interface Sci..

[20] Rice-Evans C.A., Miller N.J., Paganga G. (1996). Structure-antioxidant activity relationships of flavonoids and phenolic acids. Free Radic. Biol. Med..

[21] Benzie I.F.F., Strain J.J. (1999). Ferric Reducing/Antioxidant Power Assay: Direct Measure of Total Antioxidant Activity of Biological Fluids and Modified Version for Simultaneous Measurement of Total Antioxidant Power and Ascorbic Acid Concentration. Methods in Enzymology.

[22] Bravo-Díaz C., Romsted L.S., Liu C., Losada-Barreiro S., Pastoriza-Gallego M.J., Gao X., Gu Q., Krishnan G., Sánchez-Paz V., Zhang Y. (2015). To Model Chemical Reactivity in Heterogeneous Emulsions, Think Homogeneous Microemulsions. Langmuir.

[23] Costa M., Losada-Barreiro S., Bravo-Díaz C., Monteiro L.S., Paiva-Martins F. (2020). Interfacial Concentrations of Hydroxytyrosol Derivatives in Fish Oil-in-Water Emulsions and Nanoemulsions and Its Influence on Their Lipid Oxidation: Droplet Size Effects. Foods.

[24] Yildizdas H.Y., Poyraz B., Atli G., Sertdemir Y., Mert K., Ozlu F., Satar M. (2019). Effects of two different lipid emulsions on antioxidant status, lipid peroxidation and parenteral nutrition-related cholestasis in premature babies, a randomized-controlled study. Pediatr. Neonatol..

[25] Mollica F., Lucarini M., Passerini C., Carati C., Pavoni S., Bonoldi L., Amorati R. (2020). Effect of Antioxidants on High-Temperature Stability of Renewable Bio-Oils Revealed by an Innovative Method for the Determination of Kinetic Parameters of Oxidative Reactions. Antioxidants.

[26] Bibi Sadeer N., Montesano D., Albrizio S., Zengin G., Mahomoodally M.F. (2020). The Versatility of Antioxidant Assays in Food Science and Safety—Chemistry, Applications, Strengths, and Limitations. Antioxidants.

[27] Silva F.A.M., Borges F., Guimarães C., Lima J.L.F.C., Matos C., Reis S. (2000). Phenolic Acids and Derivatives:  Studies on the Relationship among Structure, Radical Scavenging Activity, and Physicochemical Parameters. J. Agric. Food Chem..

[28] Siquet C., Paiva-Martins F., Lima J.L.F.C., Reis S., Borges F. (2006). Antioxidant profile of dihydroxy-and trihydroxyphenolic acids-A structure–activity relationship study. Free Radic. Res..

[29] Galato D., Ckless K., Susin M.F., Giacomelli C., Ribeiro-do-Valle R.M., Spinelli A. (2001). Antioxidant capacity of phenolic and related compounds: Correlation among electrochemical, visible spectroscopy methods and structure–antioxidant activity. Redox Rep..

[30] Blasco A.J., González Crevillén A., González M.C., Escarpa A. (2007). Direct electrochemical sensing and detection of natural antioxidants and antioxidant capacity in vitro systems. Electroanalysis.

[31] Robbins R.J. (2003). Phenolic Acids in Foods:  An Overview of Analytical Methodology. J. Agric. Food Chem..

[32] Barclay L.R.C., Vinqvist M.R., Rappoport Z. (2003). Phenols as Antioxidants. The Chemistry of Phenols.

[33] Litwinienko G., Ingold K.U. (2007). Solvent Effects on the Rates and Mechanisms of Reaction of Phenols with Free Radicals. Acc. Chem. Res..

[34] Leopoldini M., Russo N., Toscano M. (2011). The molecular basis of working mechanism of natural polyphenolic antioxidants. Food Chem..

[35] Dangles O., Dufour C., Tonnelé C., Trouillas P. (2017). The Physical Chemistry of Polyphenols. Recent Adv. Polyphen. Res..

[36] Chiorcea-Paquim A., Enache T.A., De Souza Gil E., Oliveira-Brett A.M. (2020). Natural phenolic antioxidants electrochemistry: Towards a new food science methodology. Compr. Rev. Food Sci. Food Saf..

[37] Rene A., Abasq M.-L., Hauchard D., Hapiot P. (2010). How do phenolic compounds react toward superoxide ion? A simple electrochemical method for evaluating antioxidant capacity. Anal. Chem..

[38] Roleira F.M.F., Siquet C., Orrù E., Garrido E.M., Garrido J., Milhazes N., Podda G., Paiva-Martins F., Reis S., Carvalho R.A. (2010). Lipophilic phenolic antioxidants: Correlation between antioxidant profile, partition coefficients and redox properties. Bioorg. Med. Chem..

[39] Garrido J., Gaspar A., Garrido E.M., Miri R., Tavakkoli M., Pourali S., Saso L., Borges F., Firuzi O. (2012). Alkyl esters of hydroxycinnamic acids with improved antioxidant activity and lipophilicity protect PC12 cells against oxidative stress. Biochimie.

[40] Foti M.C., Daquino C., Geraci C. (2004). Electron-Transfer Reaction of Cinnamic Acids and Their Methyl Esters with the DPPH• Radical in Alcoholic Solutions. J. Org. Chem..

[41] Ordoudi S.A., Tsimidou M.Z., Vafiadis A.P., Bakalbassis E.G. (2006). Structure−DPPH• scavenging activity relationships: Parallel study of catechol and guaiacol acid derivatives. J. Agric. Food Chem..

[42] Dunford H.B. (1987). Free radicals in iron-containing systems. Free Radic. Biol. Med..

[43] Paiva-Martins F., Gordon M.H. (2002). Effects of pH and ferric ions on the antioxidant activity of olive polyphenols in oil-in-water emulsions. J. Am. Oil Chem. Soc..

[44] Paiva-Martins F., Gordon M.H. (2005). Interactions of Ferric Ions with Olive Oil Phenolic Compounds. J. Agric. Food Chem..

[45] Trujillo M., Gallardo E., Madrona A., Bravo L., Sarria B., Gonzalez-Correa J.A., Mateos R., Espartero J.L. (2014). Synthesis and antioxidant activity of nitrohydroxytyrosol and its acyl derivatives. J. Agric. Food Chem..

[46] Costa M., Losada-Barreiro S., Paiva-Martins F., Bravo-Díaz C. (2016). Optimizing the efficiency of antioxidants in emulsions by lipophilization: Tuning interfacial concentrations. RSC Adv..

