# Supplementary material for: Effects of the Reactive Moiety of Phenolipids on Their Antioxidant Efficiency in Model Emulsified Systems
Source: Foods. 2021 May 10;10(5):1028. doi: 10.3390/foods10051028 (PMC8150906; doi:10.3390/foods10051028)
Supplement: Supplementary file 1 [file foods-10-01028-s001.zip › foods-1106914-supplementary.pdf]

Effects of the reactive moiety of phenolipids on their antioxidant efficiency

Marlene Costa[1], Fátima Paiva-Martins[1]*, Sonia-Losada Barreiro[1,2], Júlia Magalhães[3], Luís S. Monteiro[4], Carlos Bravo-Díaz[2]

1) REQUIMTE/LAQV, Department of Chemistry and Biochemistry, Faculty of Sciences, University of Porto, Campo Alegre 687, Porto, Portugal

2) Department of Physical-Chemistry, Faculty of Chemistry, University of Vigo, 36310 Vigo, Spain

3) REQUIMTE/LAQV, Department of Chemical Engineering, Faculty of Engineering, University of Porto, Dr. Roberto Frias, 4200-465, Porto, Portugal

4) Chemistry Centre, University of Minho, Gualtar, 4710-057 Braga, Portugal

S1. ^{1}H and ^{13}C NMR chemical shifts for 4-hydroxycinnamic acid derivatives

S1.1. *Trans* Octyl 3-(4-hydroxyphenyl) propenoate (HCA8)

^{1}H NMR ($CDCl_3$, 400 MHz): δ 0.87 (t, J = 6.8 Hz, 3H, H-8'); 1.27 (bs, 10H, H-7'-3'); 1.68 (m, 2H, H-2'); 4.18 (t, J = 6.6 Hz, 2H, H-1´), 6.29 (d, J = 16.0 Hz, 1H, H-2), 6.86 (d, J = 8.8 Hz, 1H- H-6, 8), 7,41 (d, J = 8.4 Hz, 2H, H-5, 9); 7.62 (d, J = 16.0 Hz, 1H, H-3).

^{13}C NMR ($CDCl_3$; 100 MHz): δ 13.8 (C-8'); 22.4 (C-7'); 25.8 (C-3'); 28.5 (C-2'); 29.0 (C-4', 5'); 31.6 (C-6'); 64.6 (C-1'); 115.7 (C-6, 8); 126.7 (C-4); 129.8 (C-5, 9); 144.5 (C-3); 157.9 (C-7); 167.8 (C-1).

S1.2.*Trans* Hexadecyl 3-(4-hydroxyphenyl) propenoate (HCA16)

^{1}H NMR ($CDCl_3$, 400 MHz): δ 0.88 (t, J = 6.8 Hz, 3H, H-16'); 1.27 (bs, 22H, H-15'-3'); 1.71 (m, 2H, H-2'); 4.20 (t, J = 6.6 Hz, 2H, H-1´), 6.31 (d, J = 16.0 Hz, 1H, H-2), 6.86 (d, J = 8.4 Hz, 1H- H-6, 8), 7.44 (d, J = 8.4 Hz, 2H, H-5, 9); 7.64 (d, J = 16.0 Hz, 1H, H-3).

^{13}C NMR ($CDCl_3$; 100 MHz): δ 14.1 (C-16'); 22.7 (C-15'); 25.9 (C-3'); 28.7 (C-2'); 29.2 (C-4', 13'); 31.9 (C-14'); 64.8 (C-1'); 115.9 (C-6, 8); 127.2 (C-4); 129.9 (C-5, 9); 144.5 (C-3); 157.8 (C-7); 167.8 (C-1).

S2. ^{1}H and ^{13}C NMR chemical shifts for tyrosol derivatives

S2.1. 4-Hydroxyphenethyl octanoate (TY8)

^{1}H NMR (400 MHz, (CD3)2CO): δ 7,11 (dd, J = 8,8, J = 4,4 Hz, 2H, H4 e H8); 6,77 (dd, J = 8,8 Hz, J =4,4 Hz, 1H, H5 e H7); 4,21 (t, J = 7,2 Hz, 2H, H1); 2,83 (t, J = 7,2 Hz, 2H, H2); 2,26 (t, J = 7,2 Hz, 2H, H1'); 1,56 (quint, J = 7,2 Hz, 2H, H2'); 1,28 (m, 8H, H3'-H6'); 0,88 (t, J = 6,8 Hz, 3H, H7').

^{13}C NMR (100 MHz, (CD3)2CO): δ 174,6 (C9); 157,9 (C6); 131,7 (C4, C8); 130,7 (C3); 117,1 (C5, C7); 66,6 (C1); 36,0 (C2); 35,7 (C1'); 33,4 (C5'); 30,7 (C3', C4'); 26,7 (C2'); 24,3 (C6'); 15,3 (C7').

S2.2. 4-Hydroxyphenethyl hexadecanoate (TY16)

^{1}H NMR (400 MHz, (CD3)2CO): δ 7,09 (dd, J = 8,8, J = 4,4 Hz, 2H, H4 e H8); 6,77 (dd, J = 8,8 Hz, J =4,4 Hz, 1H, H5 e H7); 4,19 (t, J = 7,2 Hz, 2H, H1); 2,83 (t, J = 7,2 Hz, 2H, H2); 2,26 (t, J = 7,2 Hz, 2H, H1'); 1,57 (quint, J = 7,2 Hz, 2H, H2'); 1,29 (m, 24H, H3'-H14'); 0,88 (t, J = 6,8 Hz, 3H, H15').

^{13}C NMR (100 MHz, (CD3)2CO): δ 174,6 (C9); 157,9 (C6); 131,7 (C4, C8); 130,7 (C3); 117,1 (C5, C7); 66,6 (C1); 36,0 (C2); 35,7 (C1'); 33,6 (C4', C13'); 26,7 (C2'); 24,3 (C14'); 15,3 (C15').

S3. ^{1}H and ^{13}C NMR chemical shifts for dihydrocaffeic acid derivatives

S3.1. Octyl dihydrocaffeate (DCA8)

^{1}H NMR (CD_3OD, 400 MHz): δ 0.88 (t, J = 6.8 Hz, 3H, H8´), 1.30 (m, 10 H, H-7'-3'), 1.58 (m, 2 H H-2'), 2.54 (t, J = 7.6 Hz, 2H, H2), 2.76 (t, J = 7.6 Hz, 2 H, H3), 4.02 (t, J = 6.8 Hz, 2H- H-1'), 6.55 (dd, J = 8,0 1.8 Hz, 1-H, 9H), 6.71 (d, J = 1.8 Hz, 1H, 5H), 6.72 (d, J = 8.0 Hz, 1H, 8H).

^{13}C NMR (CD_3OD, 100 MHz): δ 15,3 (C-8'); 24,3 (C-7'); 27,6 (C-3'); 30,5 (C-2'); 30,9 (C-4',5'); 33,5 (C-6'); 37,8 (C-2); 65,7 (C-1'); 117,0 (C-8); 117,2 (C-5); 121,9 (C-9); 134,4 (C-4); 145,2 (C-7); 146,8 (C-6); 174.1 (C-1).

S3.2. Hexadecyl dihydrocaffeate (DCA16)

^{1}H NMR (CD_3OD, 400 MHz): δ 0,88 (t, J = 6.8 Hz, 3H, H-16'), 1.29 (m, 26H, H-15'-3'), 1,58 (m, 2H, H-2'), 2,53 (t, J = 7,6 Hz, 2H, H2), 2,76 (t, J = 7,60 Hz, 2 H-H3), 4,02 (t, J = 6,8 Hz, 2 H-H1'), 6,55 (dd, J = 8,0, J =1,8 Hz, 1H, H-9), 6,71 (d, J = 1,8 Hz, 1 H-5), 6,72 (d, J = 8,0 Hz, 1 H, H-8).

^{13}C NMR (CD_3OD, 100 MHz): δ 15,3 (C-16'); 24,3 (C-15'); 27,6 (C-3'); 30,3 (C-2'); 31,4 (C-4',13'); 32,1 (C-3); 36,6 (C-14'); 37,8 (C-2); 65,7 (C-1') 117,0 (C-8), 117,2 (C-5), 121,3 (C-9); 134,4 (C-4), 145,2 (C-7); 146,8 (C-6); 174,1 (C-1).

S4. Determination of the observed rate constant, k_{obs}, for the reaction between 16-ArN_2^+ and the antioxidants (AOs) in emulsified systems

The reaction between 16-ArN_2^+ and AOs in each region of emulsified systems is the product of the second-order rate constant and the concentration of each reactant in that region in moles per liter of region volume. 16-ArN_2^+ has a long hydrophobic alkyl chain and a cationic headgroup and is both water and oil insoluble. Its reactive -N_2^+ group is located in the interfacial region, where it reacts with AOs, as illustrated in Figure 1 and its concentration in the oil and water regions can be considered negligible. Under pseudo-first order conditions (being [AO] much higher than [16-ArN_2^+]), the observed rate is given by Equation (S1).

$$rate = k_{obs}\left[16 - ArN_{2T}^{+}\right] = k_2\left[16 - ArN_{2T}^{+}\right]\left[AO\right] = k_I\left(16 - ArN_{2I}^{+}\right)\left(AO_I\right)\Phi_I \quad \text{(S1)}$$

In Equation (S1), k_2 and k_I are the observed second rate constants and the second order rate constant in the interfacial region, respectively; k_{obs} is the observed overall rate; square brackets [], denote the concentration in mol/L of the total emulsion volume; the subscript T stands for total; parentheses (), denote the concentration in mol/L of the volume of a particular region; subscript I stands for the interfacial region; and Φ_I is the emulsifier volume fraction defined as the ratio of the volume of emulsifier divided by the total volume of the emulsion ($\Phi_I = V_{surf}/V_{Total}$).

The reaction between 16-ArN_2^+ andphenolipids, Scheme S1, was followed spectrometrically by employing the dye derivatization method (azo dye formation) described in detail elsewhere [2]. The methodology exploits the rapid reaction of 16-ArN_2^+ ions with a suitable coupling agent such as *N*-(1-

naphthyl)ethylenediamine dihydrochloride, (NED), yielding a stable azo dye whose absorbance can be determined spectrometrically at λ = 572 nm, after dilution with an alcoholic mixture. Solutions of the coupling reagent (NED) were prepared in a 50:50 (v/v) BuOH:EtOH mixture to give [NED] = 0.02 M. k_{obs} Values were obtained by fitting the absorbance-time pairs of data to the integrated first order Eq. S2.In Eq. S2, A_t, A_o and A_{inf} are the measured absorbance at any time, at t = 0 and at infinite time, respectively. An illustrative kinetic plot for the reaction between 16-ArN_2^+ and DCA8 in olive oil-in-water emulsions is shown in Figure S1. Similar kinetic plots were obtained using fish oil-in-water emulsions.

$$\ln\left(A_t - A_{\inf}\right) = \ln(A_0 - A_{\inf}) - k_{obs}t \quad \text{(S2)}$$

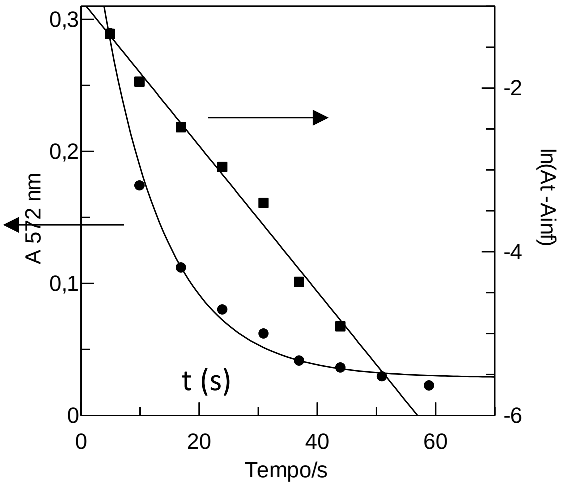

Figure S1. Illustrative variations of absorbance of azo dye from the reaction between chemical probe and NED with time in olive oil-in water emulsions containing DCA8. Experimental conditions: 4:6 (O/W, vol:vol) olive oil emulsion, Φ_I = 0.0049, [DCA8] = 4 mM, [16ArN_2^+] = 0.29 mM, citrate buffer 0.04M, pH 3.65, T = 25ºC.

S5. Determining the partition constants for water-insoluble and oil-insoluble AOs in emulsified systems

For oil-insoluble AOs, i.e., AOs that distribute between the aqueous and interfacial region such as HCA and DCA, only the constant partition P_W^I is required to define their distribution and equation S3. For

water-insoluble AOs, that is, AOs that distribute between the oil and interfacial regions such as phenolipids with alkyl chain length 8 and 16, only the P_O^I partition constant is required (equation S4). Equations S3 and S4 predict that the k_{obs} values should decrease asymptotically with the increase of Φ_I . P_W^I and P_O^I and the interfacial rate constant (K_I) were evaluated from the slopes and intercepts of the linear fits of $1/k_{obs}$ *vs* Φ_I to the reciprocals of equations S3 and S4.

$$k_{obs} = \frac{k_I[AO]_T P_W^I}{\Phi_I P_W^I + \Phi_W} \quad (S3) \qquad k_{obs} = \frac{k_I[AO]_T P_O^I}{\Phi_I P_O^I + \Phi_O} \quad (S4)$$

Figure 2S shows a typical plot of k_{obs} *vs* Φ_I in emulsions. In all cases, k_{obs} decreases by a factor of ~3-10 times by increasing the emulsifier fraction from $\Phi_I = 0.005$ to $\Phi_I = 0.04$ consistent with equations S3 and S4. The solid lines are the theoretical curves obtained by fitting the experimental data to the equations derived of the pseudophase kinetic model.

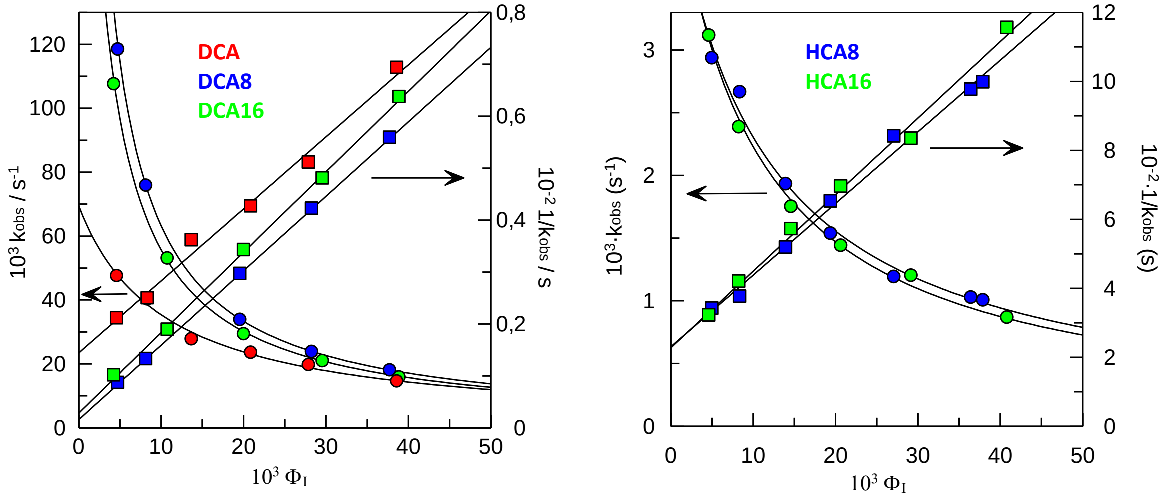

Figure S2: Representation of k_{obs} and $1 / k_{obs}$ *vs.* fraction of emulsifier (Φ_I). Experimental conditions: 4: 6 emulsion (olive oil/ citrate buffer 0.04 M, pH = 3.65) / Tween 20), [16-ArN_2^+] = 2.90x10 -4 M, [AO] = 4.00x10^{-3} M, [NED] = 0.019 M, T = 25.0.

S6. Distribution of phenolipids between oil, interfacial and aqueous regions of olive oil emulsions.

The percentage of AOs in the interfacial region, $\%AO_I$, was determined by employing Eqs. S5-S6. Equation S5 is used when the solubility in the oil region is negligible and equation S6 when the solubility in the aqueous region is negligible. Details of the calculations as well as the equations to calculate the percentages of AO in the oil and aqueous regions are given elsewhere.[11] Figure S3 shows the percentage of series of homologous antioxidants of different hydrophobicity in the interfacial region in olive oil 4:6 (O/W) emulsions.

$$\%AO_I = \frac{100\Phi_I P_W^I}{\Phi_I P_W^I + \Phi_W} \qquad \text{(S5)}$$

$$\%AO_I = \frac{100\Phi_I P_O^I}{\Phi_I P_O^I + \Phi_O} \qquad \text{(S6)}$$

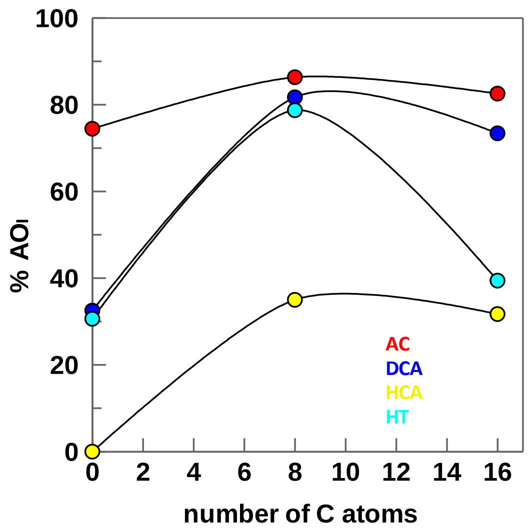

Figure S3: Percentage of AO in the interface region of emulsions 4: 6 (oil / citrate buffer, 0.04M, pH = 3.65/ Tween 20) as a function of the number of carbon atoms in the alkyl chain, for each family, for the fraction of emulsifier, Φ_I = 0.005.

S7 - Effects of the AO at selected Φ_I on the lipid oxidation reaction kinetic in olive oil in water emulsions.

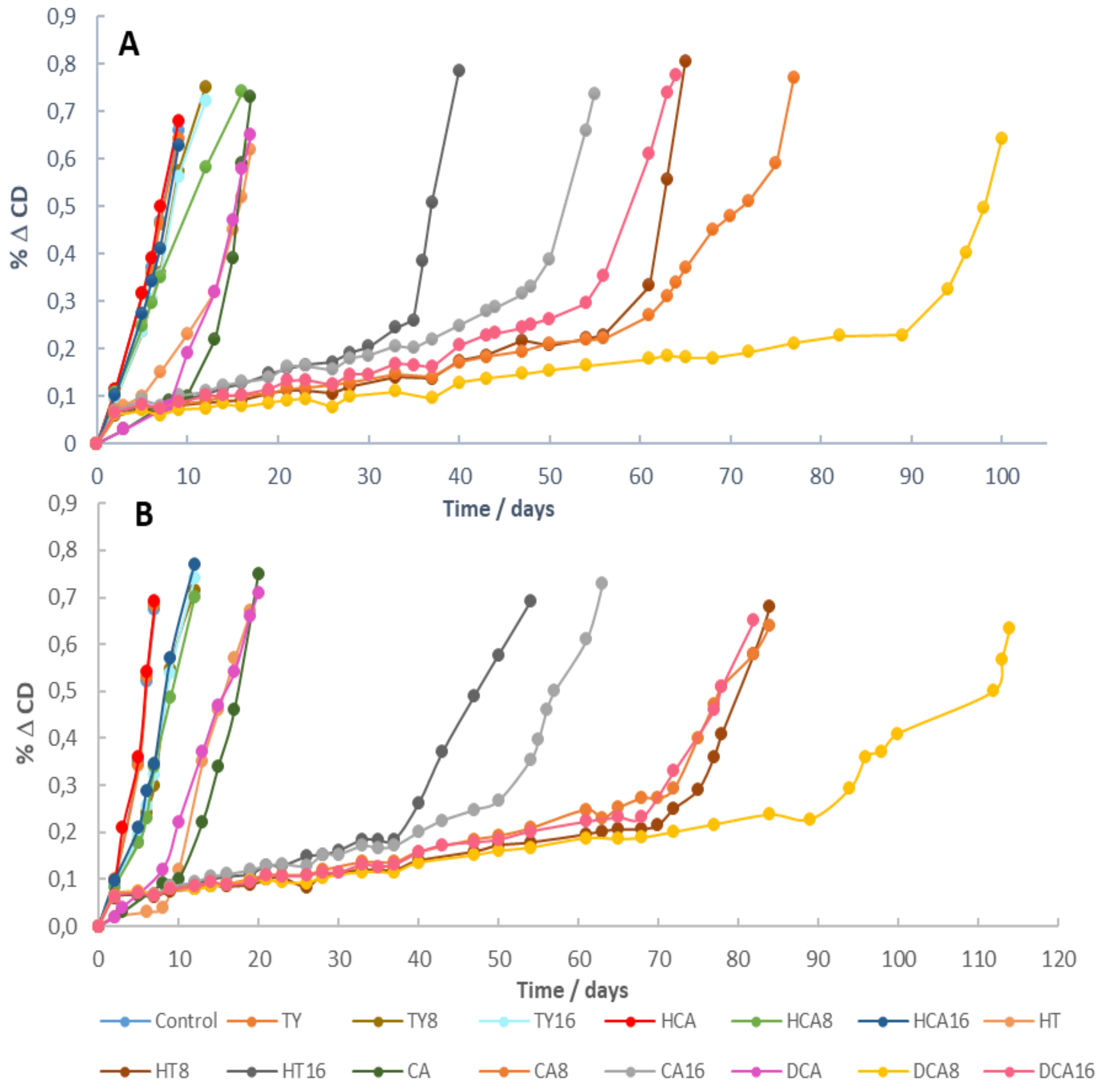

Figure S4 - Effects of the AO on the lipid oxidation reaction kinetic of 4:6 (O/W) emulsions (olive oil/citrate buffer, 0.04M, pH = 3.65/Tween 20) prepared with emulsifier fractions of Φ_I = 0.01 (A) and 0.005 (B). Mean of triplicate stored samples ($[AO_T]$ = 0.24 mM, T = 60 ºC).

S.7 Stepwise linear regression analysis (SLRA)

Table S1- Stepwise linear regression analysis Coefficients

Regression

Regression - Coefficients - Janeiro 18, 2021

Coefficients[a]

Model		Unstandardized Coefficients		Standardized Coefficients			95,0% Confidence Interval for B		Correlations			Collinearity Statistics	
		B	Std. Error	Beta	t	Sig.	Lower Bound	Upper Bound	Zero-order	Partial	Part	Tolerance	VIF
1	(Constant)	-3,302	1,460		-2,262	,035	-6,347	-,257					
	Conc.Interface	379,518	59,258	,820	6,404	,000	255,907	503,128	,820	,820	,820	1,000	1,000
2	(Constant)	,975	2,841		,343	,735	-4,972	6,922					
	Conc.Interface	334,033	62,358	,722	5,357	,000	203,516	464,549	,820	,776	,654	,822	1,217
	Ep	-7,156	4,144	-,233	-1,727	,100	-15,829	1,517	-,537	-,368	-,211	,822	1,217
3	(Constant)	7,065	3,009		2,348	,031	,743	13,386					
	Conc.Interface	229,547	60,687	,496	3,782	,001	102,049	357,045	,820	,665	,379	,584	1,713
	Ep	-13,078	3,870	-,425	-3,379	,003	-21,209	-4,947	-,537	-,623	-,338	,634	1,578
	Conc.Water	-19163,009	5989,239	-,392	-3,200	,005	-31745,934	-6580,084	-,470	-,602	-,320	,667	1,500

a. Dependent Variable: Oxidative_stability
